# Special Care Patients and Caries Prevalence in Permanent Dentition: A Systematic Review

**DOI:** 10.3390/ijerph192215194

**Published:** 2022-11-17

**Authors:** Miguel Ramón Pecci-Lloret, María Pilar Pecci-Lloret, Francisco Javier Rodríguez-Lozano

**Affiliations:** Special Care in Dentistry and Gerodontology Unit, Meseguer Hospital, Faculty of Medicine, University of Murcia, 30100 Murcia, Spain

**Keywords:** caries prevalence, permanent teeth, special needs

## Abstract

Due to the increase in the population with special needs and the significant difficulty in their dental management, it is essential to analyze the caries prevalence in this group of patients. The systematic review was conducted following the PRISMA statement. A search was performed on 9 May 2022 and updated on 5 June 2022, in three databases: Pubmed, Scielo, and Cochrane library. Studies involving the analysis of caries in permanent teeth in patients with special needs were included. A total of 1277 studies were analyzed and 21 studies were selected. Quality assessments were performed using an adapted version of the STROBE guidelines. Among the analyzed groups (intellectual disabilities, human immunodeficiency virus infection, schizophrenia, down syndrome, drug addicts, adult heart transplant, kidney disease, diabetic, autism, psychiatric patients, cerebral palsy, and hemophilia), the highest prevalence of caries was observed in patients with intellectual disability, without differences between genders. However, there is a need for more studies with standardized methods for caries diagnosis to further investigate the prevalence of caries in permanent teeth in patients with special needs.

## 1. Introduction

The concept of patients with “special needs” includes any individual whose physical, intellectual, social, or emotional abilities fall outside what is viewed as typical concerning development and standards [1]. This idea may not coincide with the classic concept of disability. Disability has sometimes been related to six specific disabilities: hearing, vision, cognition, mobility, self-care, and independent living. It is worth highlighting that 25.7% of non-institutionalized adults in the USA reported a disability [2].

The increase in the number of adults with dependency-inducing disabilities can be explained by the increase in the population over 65 years of age. This age group presents the higher prevalence of disabilities, which can diminish their life expectancy and quality of life [3].

People with disabilities often have associated oral complications. This is related to the difficulty they can experience when eating, especially when chewing, and maintaining proper oral hygiene; which can increase the susceptibility of their teeth to decay and their gums to swelling [4].

Dental caries is a multifactorial disease that involves interactions between the tooth structure, the microbial biofilm formed on the tooth surface, sugars, and salivary and genetic factors [5].

People with severe disabilities tend to have a high number of carious lesions. This may be related to difficulties in providing accessible oral health care and treatments, and with the patients’ socioeconomic context [6,7,8].

Among the main factors that participate in the increased cariogenic risk, the following are worth mentioning: xerostomia, secondary to the consumption of anxiolytics and anticholinergic drugs; the consumption of drugs that incorporate sugary vehicles; special diets, including those that require frequent intakes; hypotonia of the cheeks, lips, or tongue; motor dysfunction of hands and arms; and demotivation due to the continuous perception of illness [4,9].

The difficulty in the management of this group of patients in the dental clinic in terms of the possible drug interactions or behavior management is another barrier. Most of the patients with physical or mental disabilities cannot find or access adapted or qualified dental services at local dental clinics or even hospitals. In addition, current dental healthcare policies do not fully address this critical oral health problem [10].

Caries prevention and control, defined as the decision making and preventive and restorative treatment strategies, require initiatives, both at an individual and a collective level. For example, to control the emergence and development of dental caries, there is a need to inform and motivate the patients and their caregivers, giving dietary advice, oral care instructions, and individualized recommendations [11].

Due to the increase in the population with special needs and the significant difficulty of their dental management, it is essential to analyze the caries prevalence in this group of patients so that public dental health programs can address their needs.

## 2. Material and Methods

This systematic review was carried out following the PRISMA 2020 protocol (Preferred Reporting Items for Systematic Reviews and Meta-Analysis) [12] for systematic reviews. It was previously registered in Open Science Framework (OSF) Registries (https://osf.io/6fv3p (accessed on 17 June 2022)).

As a guide for the study of the systematic review, the following question is posed: “What is the prevalence of caries in permanent teeth in patients with special needs?”.

### 2.1. Inclusion Criteria

Articles were included according to the following criteria: (I) published in English; (II) patients with all permanent teeth; (III) patients with special needs; (IV) measurement of caries prevalence in permanent teeth; (V) specified the type of special need in relation to caries; and (VI) published in the last 10 years.

Exclusion criteria were the following: (I) languages other than English; (II) measurement of caries prevalence in deciduous teeth; (III) no measurement of caries prevalence; (IV) no relation of caries with the type of special need; (V) systematic reviews; (VI) meta-analysis; and (VII) literature reviews.

### 2.2. Search Strategy

#### 2.2.1. Databases

An initial bibliography search was conducted on 9 May 2022 and updated on 5 June 2022 on the MEDLINE database using the PubMed search engine, Scielo, and Cochrane library.

#### 2.2.2. Search Terms

The search was conducted by MPPL, it included 5 mesh (Medical Subject Heading) terms: “Caries”, “Special needs”, “Patient special needs”, “Mentally special needs” and “Physical special needs” related to each other with the Boolean operators “OR” and “AND” that were used to join the search terms: (Caries) AND ((Special needs) OR (Patient special needs) OR (Mentally special needs) OR (Physical Special Needs)) (Table 1).

#### 2.2.3. Study Selection

Following the inclusion and exclusion criteria, the articles from the last eleven years (from 2012 to 2022) were selected using the Endnote X9.3.1 manager (Clarivate, London, UK). The titles and abstracts of 1266 articles were analyzed by MRPL and MPPL after discarding duplicates. If the information was inconclusive, the full text was read. In addition, a manual search of the selected studies was carried out to find additional eligible studies.

#### 2.2.4. Data Extraction

For the bibliometric analysis, the years of publication, the city, and the journals were considered. For the synthesis of the methodology of the included studies, a summary table was made with the following data: type of special need, study type, samples, groups, mean age of participants, gender, type of caries evaluation, DMFT, decayed, caries prevalence, and comparison of caries with the control group.

### 2.3. Quality Evaluation

Two investigators (M.P.P.-L. and F.J.R.-L.) performed the quality assessment of the included studies using an adapted version of the “Strengthening the Reporting of Observational Studies in Epidemiology” (STROBE) guidelines [11]. All included studies were scored according to ten specific criteria obtained from items 5, 6, 7, 8, 10, 12, and 15 from the original checklist. Each item was scored as positive (✓) when the requirement was met and negative (✕) when not fulfilled.

After the individual assessment by each investigator, studies with 8 to 10 points were categorized as low risk of bias, 5–7 were considered a moderate risk, and those with 4 or less were selected as high risk of bias.

Final study scores for each rater were collected and scrutinized for discrepancies. A consensus decision resolved any disagreement between raters. Once consensus was reached for all study ratings, overall quality scores were collected by adding these criteria, with the maximum score being 10.

## 3. Results

### 3.1. Study Selection and Flow Diagram

**Figure 1:**
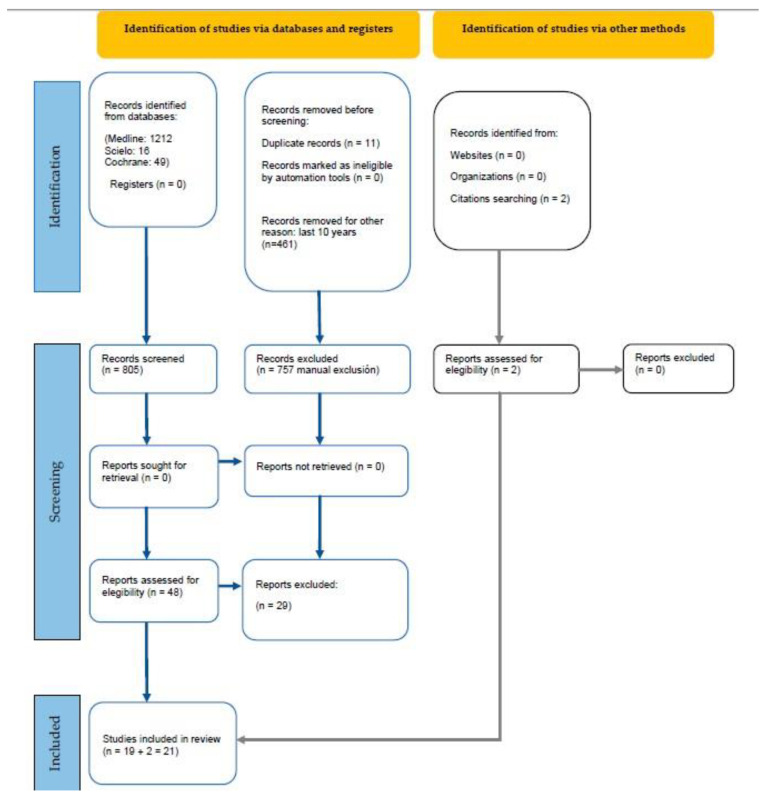
Systematic flow diagram representing the inclusion of studies according to the PRISM2A 2020 Declaration.

The search found 1277 preliminary references related to adults with special needs and caries: 1212 from Medline, 16 from Scielo, and 49 from Cochrane. After discarding 11 duplicate studies and 461 for being published more than 10 years ago, the remaining 805 records were analyzed. After analyzing the title and abstract of the records, 686 studies were excluded since they did not fit the inclusion criteria. The full texts of the remaining 48 articles were analyzed, from which 29 were excluded: 12 because they did not specify the disease; 3 because they were a review; 1 because it was a systematic review; 1 because it was in German; 8 because they analyzed caries in permanent and deciduous dentition at once; and 4 because they did not specify if patients had carious lesions. Two additional studies were found by citation searching and met the inclusion criteria. Finally, 21, articles were selected for their qualitative analysis (Figure 1).

### 3.2. Study Characteristics

#### 3.2.1. Bibliometric Analysis

**Figure 2:**
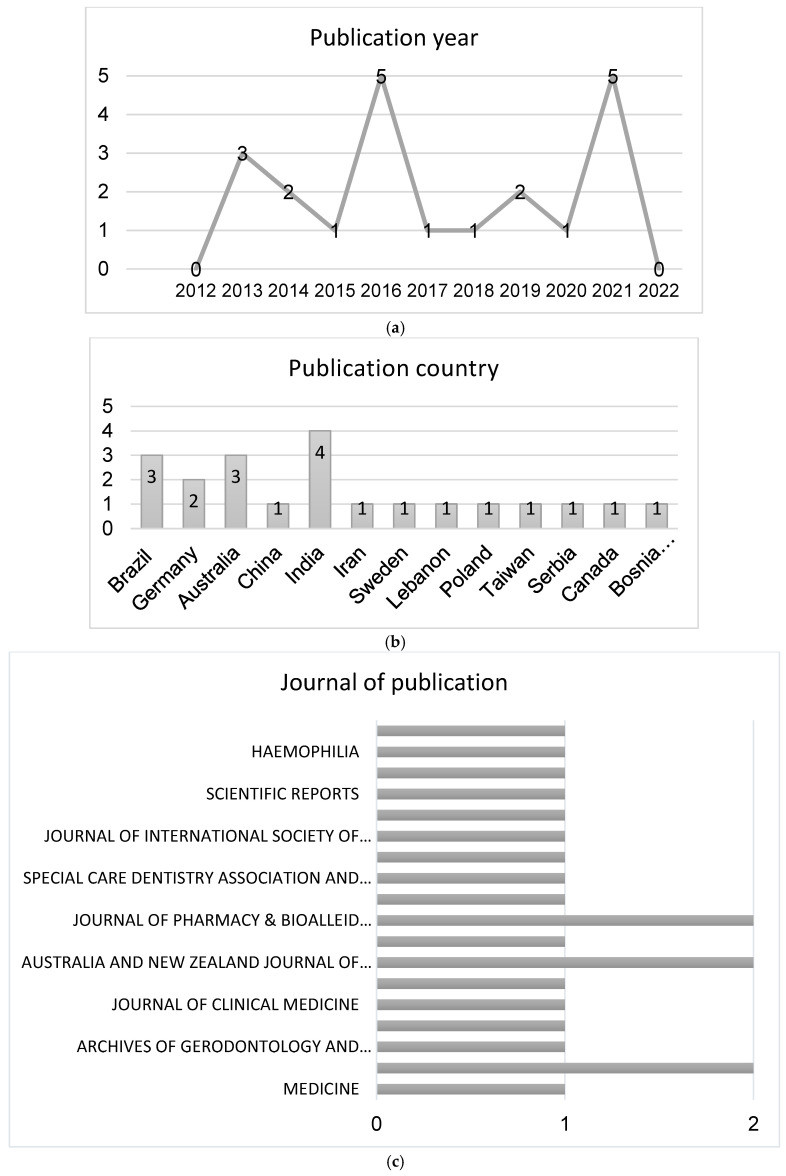
Bibliometric analysis: distribution of included studies by year of publication (**a**), country (**b**), and journal (**c**).

The maximum number of publications was observed in 2016 and 2021 (five articles per year), followed by 2013 (three articles), and 2014 and 2019 (two articles per year). Lastly, only one publication was observed in 2015, 2017, 2018, and 2020. A slight increase in the number of publications regarding this matter has been observed in the last years. Interestingly, less articles were published in 2020 compared with 2019 and 2021. This may be explained by the coronavirus disease (COVID) outbreak, where patients with special needs were considered as a group at risk. The number of patients with special needs is increasing with the increase in life expectancy; therefore, an increase in the number of this type of article is expected in the coming years (Figure 2).

Regarding the countries of publication, the highest productivity can be seen in the continent of Asia with eight publications: India has four publications; China has one publication; Iran has one publication; Lebanon has one publication; and Taiwan has one publication. Europe has six publications: two publications in Germany; one in Sweden; one in Poland; one in Serbia; and one in Bosnia and Herzegovina. America has four publications: three publications in Brazil and one publication in Canada. Finally, Oceania has three publications in Australia (Figure 2).

The distribution of publication by journals was heterogeneous. Only three journals repeated two times: “*BMC Oral Health*”, “*Australia and New Zealand Journal of Psychiatry*”, and “*Journal of Pharmacy & BioAllied Sciences*”. From the twenty-one publications selected, six journals were about oral health (*Community Dental Health*, *Archives of Gerodontology and Geriatrics*, *BMC Oral Health*, *International Journal of Dental Hygiene*, *Special Care Dentistry Association*, and Wiley Periodicals, *International Dental Journal*) (Figure 2).

#### 3.2.2. Study Types

Two studies were “case-control” and nineteen were “cross-sectional” (Table 2).

#### 3.2.3. Sample and Groups

The selected studies involved human participants: 23,114 patients were analyzed. The sample size was heterogeneous between studies. The study with fewer patients was Porovic et al. [13], with 33 patients. The study by Fernandez et al. [14] had the highest quantity of “case” patients (3525).

#### 3.2.4. Type of Patients with Special Needs

Among the group of patients with special needs, the highest number were those with intellectual disabilities, being studied 8 times [14,15,16,17,18,19,20], followed by human immunodeficiency virus infection (3 times) [21,22,23], and schizophrenia [24,25,26] and down syndrome [1,13] (2 times each). Finally, other groups of patients with special needs were only studied once: drug addicts [27], adult heart transplant [28], kidney disease [29], diabetic [30], autism [31], psychiatric patients [32], cerebral palsy, and hemophilia [26] (Table 2).

#### 3.2.5. Caries Diagnosis

From the twenty-one selected studies, only two studies [21,31] used radiographies to diagnose caries, and two used a self-reported diagnosis via a survey [15,29]. All studies performed an oral examination except one, which was self-reported [23].

#### 3.2.6. Caries Prevalence

Nine studies compared the prevalence of caries in patients with special needs with that of healthy patients [15,17,21,25,26,27,28,29,31] and found a difference between drug addicts and patients with an intellectual disability and healthy patients. However, patients with autism, heart transplant, kidney disease, hemophilia, schizophrenia, and HIV did not exhibit any difference in the DMFT (decayed, missing, and filled teeth) index compared with healthy patients. Another study on patients with an intellectual disability did not find differences from healthy patients. Only four studies [16,18,19,20] analyzed the differences between gender in caries experience, and only Ningrum et al. [16] found differences in patients with intellectual disabilities: male patients exhibited a higher number of carious lesions.

The highest DMFT scores were observed in diabetic patients [30] (27.1 and 25.8), followed by schizophrenia patients [25] (20.5), HIV patients [22,23] (15.14 and 15.6), and 14.9 in autism patients [31]. The highest decayed scores were observed in intellectual disability patients [16,17] (7.55 and 5.52) and HIV patients [17] (6.86 and 5.36). The highest prevalence of caries was observed in patients with intellectual disability [19,20] (89.4% and 95.5%), followed by psychiatric patients [32] (87.3%), and schizophrenia [25] (82.2%).

**Table 2:** Summary of Main Results of Included Studies.

Author and Year	Type of Special Patient	Study Type	Sample and Groups	Gender and Relation with Caries	Age (Mean Age) and Relation with DMFT	Caries Evaluation	DMFT and Decayed Teeth	Caries Prevalence	More Caries than HP
Agarwal et al., 2021 [24]	Schizophrenia	CS	111	M: 74.4% F: 25.3% N.I.	34.73 y DMFT increase with age	OE	M: 3.0 F: 3.1 All: 3.09 **Decayed** N.I.	N.I.	N.I.
Arora et al., 2019 [27]	Drug addicts	Case- Control	**Case**: 100 **Control**: 100	**Case**: 100 male **Control**: 100 male N.I.	**Case**: 30.8 y **Control**: 29.8 y N.I.	OE	Case: 5.71 Control 2.45 **Decayed** N.I.	N.I.	Yes
Blomqvist et al., 2015 [31]	Autism spectrum disorder	CS	Autism: 47 Control: 69	M: 53.2% F: 46.8% N.I.	33 y N.I.	OE, RE	Autism: 14.9 Control: 15.9 **Decayed:** Autism: 4.9 Control: 10.3	N.I.	No
Cao et al., 2018 [28]	Adult heart transplant (AHT)	CS	**AHT**: 81 **Control**: 63	**AHT** M: 69.1% F: 30.9% **Control** M: 63.5% F: 36.5% N.I.	**AHT**: 47.7 **Control**: 49.6 N.I.	OE	**AHT**: 3 **Control**: 3	**AHT**: 80.2% **Control**: 79.4%	No
Diab et al., 2017 [15]	Intellectual disabilities	CS	**Case**: 510 **Survey**: 1877	N.I.	Age brackets from 12 to 44 DMFT increase with age	ID: OE Survey: SR	12 y: 4.27 15 y: 4.80 35–44 y: 12.71 **Decayed:** ID 12: 3.90 15: 4.01 35–44: 3.17 Survey: 12: 5.14 15: 6.80 35–44: 7.20	N.I.	No
Fernandez et al., 2016 [14]	Intellectual disabilities	CS	Poland: 1549 Romania: 1683 Slovenia: 293	Poland: M: 69% F: 31% Romania: M: 60% F: 40% Slovenia: M: 64% F: 36% N.I.	Poland: 23.2 y Romania 22.9 y Slovenia 27.8 y N.I.	OE	N.I.	Poland: 41% Romania: 19% Slovenia 61%	N.I.
Goud et al., 2021 [32]	Psychiatric patients	CS	150	M: 60% F: 40%	33.79 y N.I.	OE	4.06 **Decayed**: 3.4	87.3%	N.I.
Kapellas et al., 2021 [29]	Kidney disease	CS	**Case**: 102 **Control**: 312 **Survey**: 4775	**Case** M: 39.2% F: 60.8% **Control** M: 55.8% F: 44.2% **Survey:** M: 49.7% F: 50.3% N.I.	**Case**: 48.1 **Control**: 40.01 **Survey**: 44.84 N.I.	**Case and control**: OE **Survey:** SR	**Case**: 0.86 **Control**: 9.72 **Survey**: 14.01 **Decayed** Case: 3.17 Control: 3.02 Survey: 0.59	N.I.	No
Liberali et al., 2013 [21]	HIV	CS	**92–93:** 54 **9–10:** 60	**92–93** M: 90.7% F: 9.3% **9–10** M: 96.7% F: 3.3% N.I.	**92–93** Age brackets from 25 to 50+ **9–10** Age brackets from 25 to 50+ N.I.	OE RE	**92–93:** 15.6 **9–10:** 8.7 AM: 15.3 **Decayed** 1992: 2.4 2009: 0.6 AM: 0.6	**92–93:** 51.9% **9–10:** 35% **Control**: 24.2%	No
Lima et al., 2019 [30]	Diabetic	CS	**Diabetics from Fortaleza:** 60 **Diabetics from Rouen:** 60	**FOR**: M: 50% F: 50% **Rouen**: M: 50% F: 50% N.I.	**Fortaleza** Age brackets from 25 to 85+ **Rouen:** Age brackets from 25 to 85+ N.I.	OE	**Fortaleza** 27.1 **Rouen**: 25.8 **Decayed** Fortaleza 0.5 Rouen 1.7	**FOR** 50% **Rouen** 38.9%	N.I.
Ningrum et al., 2020 [16]	Intellectual disability	CS	65	M: 48% F: 52% **Male**	22 y N.I.	OE	N.I. **Decayed** F: 3.00 M: 7.55	N.I.	N.I.
Oliveira et al., 2013 [17]	Intellectual disability	CS	**ID**: 103 **Siblings**: 103	ID: M: 60% F: 40% Siblings M: 40% F: 60% N.I.	Age brackets from 12 to 36 N.I.	OE	ID: 6.36 Siblings: 5.06 **Decayed:** ID: 3.52 Siblings: 2.03	ID: 55.3% Siblings: 40.1%	Yes
Petrovic et al., 2016 [18]	Intellectual disability	CS	726	M: 62.75% F: 37.25% No differences between gender	Age bracets from 12 to 20+ No differences between age	OE	12–20: 3.89 +20: 4.83 M: 4.73 F: 3.90 **Decayed:** 4.37	N.I.	N.I.
Pini et al., 2016 [1]	Down syndrome Cerebral palsy Intellectual disability	CS	DS: 17 CP: 13 ID: 17	DS M: 41% F: 59% CP M: 69% F: 31% ID M: 59% F: 41% N.I.	Age brackets from 12 to 55 N.I.	OE	DS: <10:47% >10: 52% CP: <10: 69% >10: 30% ID: <10 53% >10: 47% **Decayed:** N.I.	N.I.	N.I.
Porovic et al., 2016 [13]	Down syndrome	CS	33	M: 63.6% F: 36.4% N.I.	From 19 to 45 N.I.	OE	15.96 **Decayed:** 3.57	N.I.	N.I.
Rezaei-Soufi et al., 2014 [22]	HIV	CS	HIV T 50 HIV WT 50	**Treatment** M: 71.4% F: 28.6% **Control** M: 81.8% F: 18.2% N.I.	**Case**: 36 y **Control**: 38 y N.I.	OE	Case: 15.14 Control: 14.45 **Decayed** Case: 6.86 Control: 5.36	**Treatment:** 34.52% **Control:** 27.42%	N.I.
Rungsiyanont et al., 2012 [23]	HIV	CS	299	M: 28.6% F: 71% N.I.	36.7 y N.I.	SR	N.I.	65.9%	N.I.
Schmidt et al., 2021 [19]	Intellectual disability	CS	132	M: 49.2% F: 51.8% No differences between gender	35.2 y DMFT increased with age	OE	All: 9.5 M:9.9 F: 9.2 **Decayed** All: 0.5 M: 0.5 F: 0.6	**All**: 89.4% **M:** 87.1% **F:** 91%	N.I.
Schulte et al., 2013 [20]	Intellectual disability	CS	428	M: 51.4% F: 48.6% No differences between gender	All: 35.5 y M: 36.7 y F: 34.3 y DMFT increased with age	OE	All:12.25 M: 12.42 F: 12.09 **Decayed** M: 2.14 F: 1.83	**All**: 95.5% **Caries free**: 4.4% **M**: 95.7% **F**: 95.4%	N.I.
Wey et al., 2016 [25]	Schizophrenia	CS	**Case**: 543 **Survey**: 8332	M: 66.7% F: 33.3% N.I.	54.8 y DMFT increased with age	Case: OE Survey: SR	Case: 20.5 Survey: 11.6 **Decayed** Case: 3.48 Survey: 1.70	**Case:** 82.2% **Survey:** 89.3%	No
Zaliunienne et al., 2014 [26]	Haemophilia	Case-Control	**Case**: 75 **Control**: 72	M: 100% N.I.	Age bracets from 11 to 60 N.I.	OE, RE	Case: 9.4 Control: 9.3 **Decayed:** Case: 3.1 Control: 2.7	N.I.	No

HIV T: HIV with treatment; HIV WT: HIV without treatment; M: male; F: female; HP: healthy patient; N.I: no information; OE: oral exploration; SR: self-reported; RE: radiograph exploration; AM: aged matched; DMFT: decayed, missing, and filled teeth; DS: down syndrome; CP: cerebral palsy; ID: intellectual disability.

### 3.3. Quality Assessment

All observational studies were analyzed using adapted STROBE guidelines for rating observational studies [33] (Table 3). One study had a score of 4, two studies had a score of 5, four had a score of 6, seven had a score of 7, five had a score of 8, and two had a score of 9 (Table 4). Three studies were classified as having a high risk of bias, five as moderate risk of bias, and three as low risk of bias.

Only criteria #10 “Measures and presents exposure data” was fulfilled by all selected studies. Criteria #9 “Provides characteristics of study participants (e.g., demographic, clinical, social) and reports on exposures and potential confounders” and criteria #3 “Describes the disease studied” were assessed by 20 of the 21 selected studies. Criteria #7 “Describes statistical methods, including those used to control for confounders” was assessed by 19 of the 21 selected studies. Criteria #2 “Gives the inclusion and exclusion criteria (including paired or control groups)” was assessed by 18 of the 21 selected studies. Criteria #1 “Describes the setting, participating locations, relevant dates (period of recruitment, exposure, follow-up, data collection)” and criteria #5 “Measure of caries by itself” were assessed by 14 of the 21 selected studies. Criteria #8 “Describes any methods used to examine subgroups and interactions, and reports on exposures and potential confounders” was assessed by 10 of the 21 selected studies. Criteria #4 “Clearly define the diagnostic criteria of caries (ICDAS, light, Rx” was assessed by 8 of the 21 selected studies. Finally, criteria #6 “Explains how the study sample size was arrived at,” was assessed by only 2 of the 21 selected studies.

## 4. Discussion

This systematic review aimed to analyze the caries prevalence in permanent teeth in patients with special needs. The increased knowledge of the groups with higher prevalence and risk of caries could be helpful to design and implement specific dental programs to help the oral health of this group of patients.

Regarding the methodology followed in the studies, the evaluation and diagnosis of caries was performed in most studies via visual examination by a dentist. However, this type of diagnosis is subjective and can differ from one professional to another. There are other types of techniques for caries detection such as quantitative light-induced fluorescence (QLF), Diagnodent (DD), fiber-optic transillumination (FOTI), electrical conductance (EC), or near-infrared imaging (NIRI) among others [34,35]. Only two studies used oral and radiographic examination [21,31], which is more specific than only oral examination for caries diagnosis. In addition, three studies used “self-reported diagnosis of caries” [23,25,29], which cannot be considered a reliable diagnostic method because the general population cannot diagnose accurately without dental training.

DMFT index is classified in five levels: 0.0–4.9 very low, 5.0–8.9 low, 9.0–13.9 moderate, 14.0–17.9 high, and more than 18 very high [36]. Seventeen of the twenty-one selected studies measured DMFT index among the sample of patients with special needs: HIV [20,21] and autism [31] had a high index (15.6, 15.14, and 14.9, respectively), and patients with diabetes mellitus [30] and schizophrenia [25] had a very high index (27.1, 25.8, and 20.5, respectively). DMFT index in autism was lower (14.9) than the control group (15.9). These results coincide with those from Corridore et al. [37]. Kapellas et al. [29] showed that the DMFT in the case group was 10.86, the control group was 9.72, and the survey group was 14.01. Still, the results from the last group were self-reported by the patient, and consequently they may not be accurate.

In the analysis of decayed teeth, Kapellas et al. [29] reported a score of 3.17 in the “case” group, 3.02 in the “control” group, and 0.59 in the survey group. The score was the highest in the group of patients with special needs, but the differences with the control group were not significant. In the case of the survey group, the decayed teeth score is less than the other two, but, again, these results should be interpreted with caution since the diagnosis was self-reported. Arora et al. [27] analyzed the drug addicts group and concluded that there were statistical differences between groups in the DMFT index (5.71 in the “case” group and 2.45 in the “control” group). This could be explained by the increased vulnerability and diminished oral-health-related quality of life in drug addicts depending on the type of drug [38], although this study had a high risk of bias. Another group of studies [19,20] analyzed the differences between the DMFT index in men and women with an intellectual disability and did not find significant differences.

The DMFT index was used in nineteen of the twenty-one studies, which is a subjective index since it does not describe how caries are identified as caries. In addition, the DMFT index does not reflect the number of active caries since it also measures absences (which can be extractions performed for other reasons, such as orthodontics or fracture, and the patient’s agenesis) and fillings. There are other indexes such as ICDAS (The International Caries Detection and Assessment System) or ICCMS (The International Caries Classification and Management System), which describe and specify when a black stain can be considered as caries [39,40,41].

Six studies analyzed the DMFT index concerning age; five concluded that DMFT increased with age, which agrees with other studies [42,43]. It could be explained because when the age rises, the number of patients with edentulism increases, increasing the DMFT index [25]. Four of the selected studies analyzed the DMFT in relation to gender; three concluded there is no relation with gender, which agrees with other authors such as Mohammadi et al. [42].

The last study of the prevalence of caries in the general population is from the World Health Organization from the year 2000. The prevalence of caries in permanent teeth is between 25% and 50% [44], being below the data obtained in some of the studies: patients with HIV with a prevalence of 65.9% [23], intellectual disability with a prevalence of 89.4% and 95.5% [19,20], psychiatric patients [32] with 87.3%, schizophrenia [25] with 82.2% and, finally, adults with a heart transplant [28] with 80.2%. However, in this study, the control group had a prevalence of 79.4%, which was higher than in the other studies. The prevalence of caries was analyzed in eleven of the twenty-one selected studies. The highest prevalence was found in the intellectual disability group [19,20] with 89.4% and 95.5%. This could be explained because this type of patient usually has the worst oral hygiene and needs their caregivers help to maintain it [45].

It should be highlighted that this study has a series of limitations: the concept of “special needs patient” is too broad, and it covers a wide range of patients with different types of needs; some of them need the help of a caregiver, others do not; and the kind of medication required for each group is different. All these variants made some groups more vulnerable to present caries than other.

## 5. Conclusions

The prevalence of caries in permanent teeth in patients with special needs seems to be higher than the general population. Based on the results of the included studies, patients with HIV present a prevalence of 65.9%, patients with intellectual disability present a prevalence of 89.4% and 95.5%, psychiatric patients 87.3%, schizophrenia 82.2% and, finally, adults with a heart transplant 80.2%. However, there is a need for more studies with standardized caries diagnosis methods to further investigate the caries prevalence in permanent teeth in patients with special needs.

## Figures and Tables

**Table 1:** Search strategy.

**Search Strategy**	**#1**	**Caries**
	**#2**	**(Special Needs) OR (Patient Special Needs) OR (Mentally Special Needs) OR (Physical Special Needs)**
	**#1 AND #2**	**(Caries) AND ((Special Needs) OR (Patient Special Needs) OR (Mentally Special Needs) OR (Physical Special Needs))**
**Database**	**Search Strategy**	**Findings**
MEDLINE	#1	66,103
	#2	431,625
	#1 AND #2	1212
SciELO	#1	2469
	#2	1091
	#1 AND #2	16
Cochrane Library	#1	7792
	#2	4145
	#1 AND #2	49

**Table 3:** Checklist of 10 criteria based on an adapted version of the STROBE guidelines for assessing the quality of observational studies.

Methods		
Configuration	1	Describes the setting, participating locations, relevant dates (period of recruitment, exposure, follow-up, data collection).
Participants	2	Gives the inclusion and exclusion criteria (including paired or control groups)
	3	Describes the disease studied.
Variables	4	Clearly defines the diagnostic criteria (ICDAS, light, Rx…).
Data/measurement sources	5	Measure of caries by itself.
Study size	6	Explains how the study sample size was arrived at.
Statistical methods	7	Describes statistical methods, including those used to control for confounders.
	8	Describes any methods used to examine subgroups and interactions.
Results		
Descriptive data	9	Provides characteristics of study participants (e.g., demographic, clinical, social) and reports on exposures and potential confounders.
Result data	10	Measures and presents exposure data.

**Table 4:** Results of quality assessment using an adapted version of the STROBE guidelines.

Items	1	2	3	4	5	6	7	8	9	10	Total Score	Risk of Bias
Agarwal et al., 2021 [24]	✓	✓	✓	✕	✕	✕	✓	✓	✓	✓	7	Moderate
Arora et al., 2019 [27]	✕	✓	✓	✕	✕	✕	✓	✕	✓	✓	5	High
Blomqvist et al., 2015 [31]	✕	✓	✓	✓	✓	✕	✓	✕	✓	✓	7	Moderate
Cao et al., 2018 [28]	✓	✓	✓	✕	✕	✕	✓	✕	✓	✓	6	Moderate
Diab et al., 2017 [15]	✓	✓	✓	✕	✓	✕	✓	✓	✓	✓	8	Low
Fernández et al., 2016 [14]	✓	✓	✓	✕	✕	✕	✕	✕	✕	✓	4	High
Goud et al., 2021 [32]	✕	✕	✓	✓	✓	✓	✓	✕	✓	✓	7	Moderate
Kapellas et al., 2021 [29]	✕	✓	✓	✕	✓	✕	✓	✕	✓	✓	6	Moderate
Liberali et al., 2013 [21]	✓	✓	✓	✓	✓	✕	✓	✕	✓	✓	8	Low
Lima et al., 2019 [30]	✕	✓	✓	✕	✓	✕	✓	✕	✓	✓	6	Moderate
Ningrum et al., 2020 [16]	✓	✓	✓	✕	✕	✕	✓	✓	✓	✓	7	Moderate
Oliveira et al., 2013 [17]	✓	✓	✓	✓	✓	✕	✓	✓	✓	✓	9	Low
Petrovic et al., 2016 [18]	✕	✕	✕	✓	✕	✕	✓	✓	✓	✓	5	High
Pini et al., 2016 [1]	✓	✕	✓	✓	✕	✕	✓	✓	✓	✓	7	Moderate
Porovic et al., 2016 [13]	✓	✕	✓	✓	✓	✕	✓	✕	✓	✓	7	Moderate
Rezaei-Soufi et al., 2013 [22]	✕	✓	✓	✕	✓	✕	✓	✕	✓	✓	6	Moderate
Rungsiyanont et al., 2012 [23]	✓	✓	✓	✕	✓	✓	✕	✕	✓	✓	7	Moderate
Schmidt et al., 2021 [19]	✓	✓	✓	✕	✓	✕	✓	✓	✓	✓	8	Low
Schulte et al., 2013 [20]	✓	✓	✓	✕	✓	✕	✓	✓	✓	✓	8	Low
Wey et al., 2016 [25]	✓	✓	✓	✕	✓	✕	✓	✓	✓	✓	8	Low
Zalumienne et al., 2014 [26]	✓	✓	✓	✓	✓	✕	✓	✓	✓	✓	9	Low

Each item was scored as positive (✓) when the requirement was met and negative (✕) when not fulfilled.

## Data Availability

Not applicable.
